# Utility of Vibration Perception Thresholds as a Biomarker of Chemotherapy‐Induced Peripheral Neuropathy: A Systematic Review and Meta‐Analysis

**DOI:** 10.1002/ejp.70319

**Published:** 2026-07-01

**Authors:** Faris Khan, Max Holcroft, James P. Dunham, Anthony E. Pickering, Marin Dujmović

**Affiliations:** ^1^ Anaesthesia, Pain and Critical Care Research Group, School of Physiology, Pharmacology and Neuroscience University of Bristol Bristol UK; ^2^ Royal Papworth Hospital NHS Foundation Trust Cambridge UK

## Abstract

**Background and Objective:**

Chemotherapy‐induced peripheral neuropathy (CIPN) is a common, debilitating, and treatment‐limiting adverse effect of many agents used for cancer treatment. There is currently no gold‐standard diagnostic criterion nor a widely accepted method for accurate and early identification of CIPN. Vibration perception threshold (VPT), which reflects large‐fibre nerve function, has been proposed as a potential biomarker for CIPN.

**Databases and Data Treatment:**

A systematic review and meta‐analysis was pre‐registered and conducted by searching PubMed, EMBASE, Scopus, CENTRAL, and Web of Science databases from inception to November 2024. The primary outcome was the change in VPT following neurotoxic chemotherapy compared to the baseline/control group. PROSPERO CRD42024584611.

**Results:**

Thirty‐one studies involving 1635 participants were included in the final analysis. There was a moderate‐to‐large increase of VPT following chemotherapy in both the hands (pooled SMD = 0.75, 95% CI [0.52, 0.97]) and feet (SMD = 0.69 95% CI [0.50, 0.88]). Heterogeneity of effects was larger in the hands (*I* ^2^ = 77.10%) than the feet (*I* ^2^ = 69.10%). Meta‐regression showed that combination treatment with taxane and platinum agents produced a greater increase in VPT than either alone. Few studies (*n* = 5) investigated VPT changes specifically in patients diagnosed with CIPN limiting the ability to assess its utility as a biomarker.

**Conclusions:**

This study demonstrates consistent increases in VPT (loss of sensitivity) following chemotherapy. Future studies should incorporate clear CIPN diagnostic criteria, comparative analyses between patients with and without CIPN, and longitudinal testing to determine the utility of VPT as an early biomarker of neurotoxicity.

**Significance:**

This review demonstrates that vibration perception threshold (VPT) consistently worsens following chemotherapy, with moderate pooled effect sizes. Comparable changes were observed in both the hands and feet, and combination therapy with taxane and platinum agents was associated with greater deterioration in vibration perception measured in the hand. Together, they support VPT as a promising objective measure that can contribute to the assessment of peripheral neurotoxicity, with hand‐based testing potentially offering a practical and sensitive site for assessment.

## 1 Introduction

Chemotherapy‐induced peripheral neuropathy (CIPN) is a prevalent and often debilitating side effect of many chemotherapeutic agents used as first‐line treatments for cancer, including platinum‐based compounds, taxanes, and vinca alkaloids (Seretny et al. 2014; Stone and DeAngelis 2016). This neuropathy typically presents in a “glove and stocking” distribution with a range of symptoms, including paraesthesia, numbness, impaired dexterity, and neuropathic pain (Staff et al. 2017). Approximately 30%–40% of patients undergoing treatment with neurotoxic chemotherapy agents will have CIPN that is still present 6 months after the end of their treatment (Seretny et al. 2014; Staff et al. 2017). Despite its prevalence, there are no preventative interventions for CIPN nor effective treatment options for the symptoms of CIPN (Jordan et al. 2020). Consequently, the development of CIPN often necessitates a reduction of chemotherapy dose/frequency of dosing or switch to less neurotoxic chemotherapy agents, which may reduce the effectiveness of the planned treatment for cancer (Flatters et al. 2017). Therefore, early identification of CIPN is important so that treatment regime alterations can be made in time to limit further neurotoxicity.

Detection of CIPN currently relies on patient reported symptoms—sometimes assessed using structured questionnaires. This can be complemented by clinical examination for signs of CIPN involving sensory testing of light touch and proprioception, vibration testing with a tuning fork and eliciting tendon reflexes. While these methods are relatively simple to deploy and widely used, they are often insensitive to the early changes of neuropathy, meaning the prevalence of CIPN is likely underestimated (Burgess et al. 2021; Seretny et al. 2014). In research settings, more formal quantitative sensory testing (QST) is often undertaken (Ekman et al. 2021; Martland et al. 2020; Weaver et al. 2022). This involves a standardised battery of stimuli delivered in a carefully controlled fashion, for example, detecting changes in temperature sensitivity and pain thresholds.

One component of QST assessment of CIPN is vibration perception threshold (VPT) which reflects the function of Aβ rapidly‐adapting primary afferents. VPT is determined by finding the lowest intensity at which a subject perceives a vibration stimulus. Vibration testing is quick, relatively simple, well tolerated, and non‐invasive, making it an attractive option for routine clinical use (Lanting et al. 2020). There are a variety of different vibration testing methods and techniques, such as the classical calibrated Rydel‐Seiffer tuning fork or haptic devices. The tuning fork is also sometimes used in clinical assessments, but this is usually not a calibrated instrument but the less quantitative standard tuning fork which provides a binary measure of absence or presence of function. However, even the calibrated Rydel‐Seiffer tuning fork has limitations when used in QST for research. This is largely due to its low sensitivity and floor effect for detecting potentially clinically relevant changes in vibration perception thresholds. For example, Dujmović et al. (2025) demonstrated that the tuning fork failed to detect changes in a model of sensory loss as well as age‐related decline, unlike more sensitive haptic‐based approaches.

VPT is known to be impaired in other peripheral neuropathies, such as diabetic neuropathy and alcohol‐related neuropathy (Ekman et al. 2021; Julian et al. 2019). Accordingly, VPT has been highlighted as a potential biomarker for CIPN (Lanting et al. 2020; Weaver et al. 2022) although its performance has not been formally synthesised across studies. The aim of this systematic review and meta‐analysis is to quantify changes in VPT following chemotherapy, to examine whether such changes are more pronounced in patients who develop CIPN compared with those who do not, and to explore the influence of factors such as chemotherapy class, testing site, measurement method, and patient characteristics. Doing so, we evaluate whether VPT represents a useful predictive biomarker of CIPN.

## 2 Methods

### 2.1 Study Protocol

This systematic review was conducted following the Preferred Reporting Items for Systematic Reviews and Meta‐Analyses (PRISMA) guidelines (Page et al. 2021). The main concepts of this research were identified to formulate a research question using the PICO (Population; Intervention; Comparison and Outcome) framework. The research question is ‘In patients with cancer (Population) undergoing chemotherapy (Intervention), compared to individuals who have not undergone chemotherapy or within‐subject baseline measurement (Comparison), is there a difference in vibration perception threshold, and can this be used as a diagnostic test for chemotherapy‐induced peripheral neuropathy (CIPN) (Outcomes)? Our review followed an a priori registered study protocol (PROSPERO 2024 CRD42024584611).

### 2.2 Search Strategy

An electronic literature search was conducted in PubMed, EMBASE, Scopus, Cochrane Central Register of Controlled Trials (CENTRAL), and Web of Science from database inception to November 2024. The search combined subject headings (MeSH/Emtree) and free‐text keywords related to vibration perception and chemotherapy‐induced peripheral neuropathy (CIPN). Search strings included terms such as “chemotherapy‐induced neuropathy”, “CIPN”, “peripheral neuropathy”, “neurotoxicity”, “vibration perception threshold (VPT)”, “vibration detection threshold (VDT)”, and “vibration testing”. The full search strategies for each database are provided in Methods S1.

### 2.3 Eligibility Criteria

#### 2.3.1 Population

Studies were eligible if they included human participants with a diagnosis of cancer of any type, who were undergoing or had previously undergone chemotherapy treatment. Participants of all ages were included. Studies were excluded if they did not involve a patient population receiving chemotherapy.

#### 2.3.2 Intervention

Eligible studies were required to report outcomes for patients treated with chemotherapy, with sufficient detail provided on the chemotherapy regimen (e.g., agent or combination). Studies were excluded if the chemotherapy exposure was not adequately described, including if the chemotherapy agents were not specified, or if the timing of chemotherapy exposure relative to outcome assessment was not described. Additional details such as dose, cumulative dose, and treatment schedule were recorded where available but were not mandatory for inclusion.

#### 2.3.3 Comparators

We included studies that reported comparison groups consisting of (a) healthy age‐ and sex‐matched controls, (b) age‐ and sex‐matched cancer patients prior to initiation of chemotherapy, or (c) within‐subject pre‐chemotherapy baseline measurements. No other restrictions were applied regarding comparator type, provided at least one of the above groups was available.

#### 2.3.4 Outcomes

Eligible studies were required to assess change in vibration perception threshold (VPT) using a validated, commercial vibration testing device. Studies were included if they reported changes in VPT over time in chemotherapy‐treated patients, differences in VPT between patients with and without CIPN, or associations between VPT and clinical or demographic factors. Studies that did not include VPT measurement as an outcome were excluded.

#### 2.3.5 Study Design

Randomised controlled trials, non‐randomised controlled trials, prospective cohort studies, cross‐sectional studies, and case–control studies were included. Studies were required to be peer‐reviewed and published in full‐text form. Review papers, case reports, case series, conference abstracts, expert opinions, and other forms of non‐peer reviewed or grey literature were excluded. Only studies published in English were included.

### 2.4 Study Selection

All records were imported into Covidence (Veritas Health Innovation, Melbourne, Australia), where duplicate records were automatically identified and removed. Two reviewers (FK, MH) independently screened titles and abstracts according to the predefined eligibility criteria. Full‐text articles were then assessed for inclusion by the same reviewers. Any disagreements were resolved through discussion, and where necessary, adjudication by a third reviewer (MD).

### 2.5 Data Extraction

Data were extracted using a predefined template in Covidence by two researchers (FK, MH). Extracted data included study characteristics, participant demographics, chemotherapy regimens, vibration sensitivity measurement methods, and outcomes. For studies where necessary data were not reported, corresponding authors were contacted to obtain missing information. None of the contacted authors provided data.

### 2.6 Data Synthesis

For our primary outcome of change in VPT from pre‐chemotherapy baseline to a defined post‐chemotherapy timepoint, we calculated the within‐participant change in VPT (as a standardised mean difference, SMD). In studies with measurements at multiple time points, the final measurement before the start of chemotherapy was selected as the baseline and the first measurement following completion of chemotherapy (or latest measurement during chemotherapy) as the comparator. When only mid‐chemotherapy data was available, the final measurement reported was taken as the post comparator. When studies reported mean change from baseline and appropriate standard error/standard deviation, we used these values directly in the meta‐analysis. For within‐participant comparisons where the authors do not provide mean changes from baseline and accompanying standard errors, we computed these from available means and standard deviations (before and after chemotherapy), while assuming a 0.3 correlation between repeated measures. We chose a conservative correlation estimate due to the heterogeneity of outcomes for chemotherapy patients. While CIPN is quite prevalent, the magnitude of impact varies considerably, and as such we would not expect a high correlation between pre‐chemo and post‐chemo measurements.

A small number of studies (*N* = 3) included healthy control groups; however, none provided data suitable for a formal between‐group meta‐analysis as none provided comparable longitudinal data for change in VPT in the control groups.

We used random‐effects models (REML) to estimate the overall standardised mean differences (SMDs). Heterogeneity was summarised with *τ* ^2^ and *I* ^2^. Influence of individual studies was evaluated with leave‐one‐out analysis and the likelihood of missing studies inferred via funnel plots.

As VPT is predominantly measured at two different sites—lower and upper limbs—we defined two co‐primary analyses: assessment in the hand and foot. We did not stratify by study design (RCT vs. non‐RCT) in the main analysis. We do not focus on the intervention in RCTs (usually an intervention aimed at reducing CIPN incidence) and extract data from pre and post‐treatment measurements in both groups. A random‐effects meta‐regression was conducted to examine whether chemotherapy type (platinum, taxane, or a combination) predicted differences in VPT.

Meta‐analysis and meta‐regression were conducted using the *meta* v8.2‐1 R package (Schwarzer et al. 2015). The extracted data and analysis scripts can be found on OSF at https://doi.org/10.17605/OSF.IO/PE7MQ.

Where studies of secondary outcomes could not be meta‐analysed due to insufficient data or excessive study heterogeneity, they were reported narratively.

### 2.7 Quality Assessment

Risk of bias was independently assessed by two reviewers (FK, MH) using the ROBINS‐I (Risk Of Bias In Non‐randomised Studies of Interventions) tool for non‐randomised studies and the ROB‐2 (Risk of Bias 2) tool for RCTs.

## 3 Results

**FIGURE 1:**
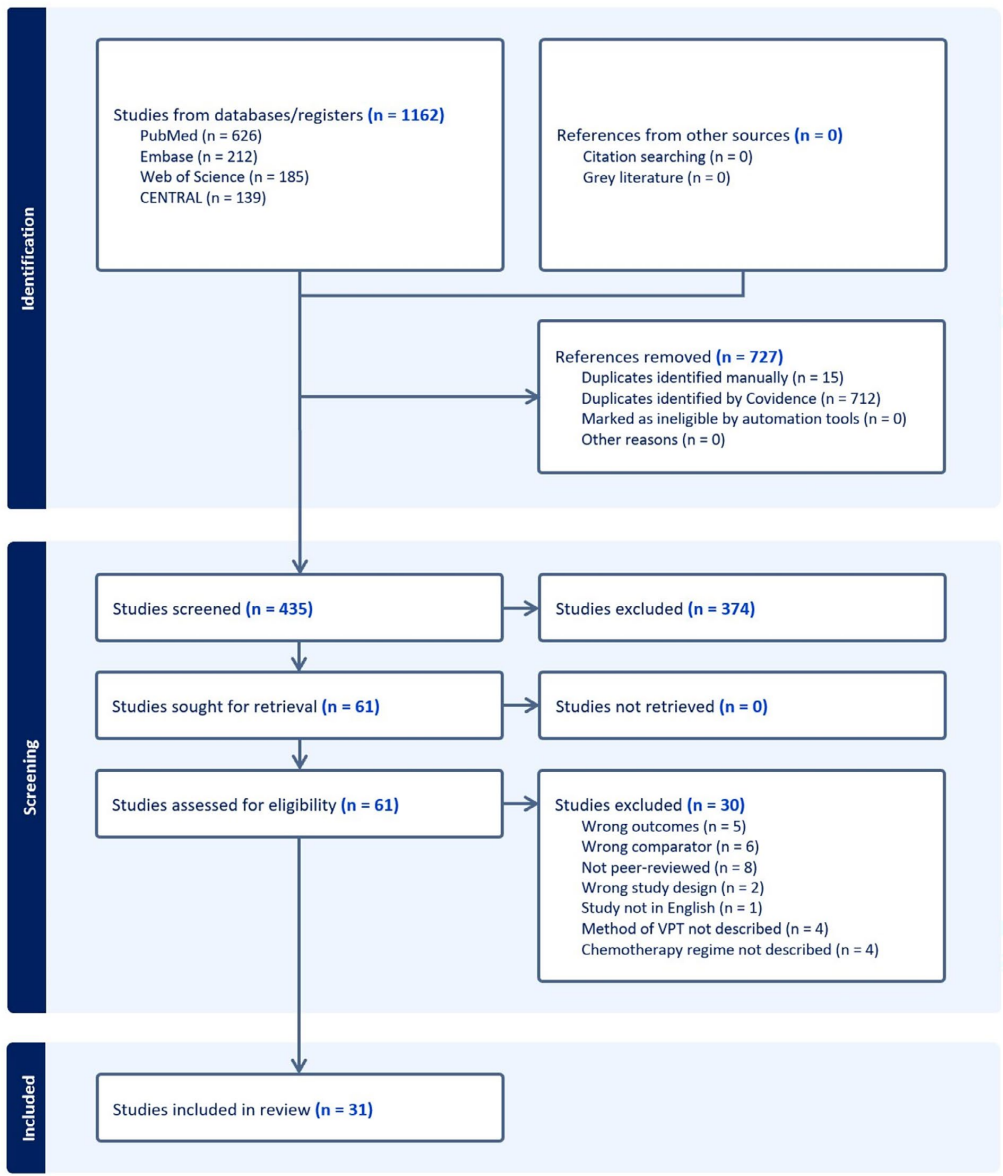
PRISMA (Preferred Reporting Items for Systematic reviews and Meta‐Analyses) flow‐chart.

The systematic search following the predefined inclusion and exclusion criteria identified 435 articles. After title screening, 61 papers were selected for full text screening. Subsequently, 31 articles were included as part of this systematic review and meta‐analysis. The PRISMA flowchart outlining the study selection process, along with exclusion reasons, is presented in Figure 1.

Most of the included studies were non‐randomised controlled trials (*n* = 25, primarily prospective cohort studies), and a minority were randomised controlled trials (*n* = 6). In total 1635 participants (1567 patients with cancer and 48 healthy controls) were included. Study sample sizes ranged from 5 to 196 patients (median 48). The most prevalent cancer type among the subjects was ovarian cancer. Cisplatin was the most common chemotherapy agent (*n* = 578), followed by Paclitaxel (*n* = 352), Oxaliplatin (*n* = 279), and Docetaxel (*n* = 160). One study did not involve a chemotherapy regime with a platinum agent and/or a taxane, and studied Ixabepilone (Goel et al. 2008). Vibration testing was performed with a variety of different devices; the most common was one of the iterations of the Vibrameter (*n* = 16) (Somedic SenseLab AB, Stockholm, Sweden). Vibration testing was performed in a hospital‐based clinical environment as a component of the patient's wider cancer treatment and monitoring. The key characteristics of the included studies and patients are detailed in Table S1.

For our primary outcome of the change in VPT following chemotherapy, specific domains of the tools are relevant to assessing bias in measurement of the outcome; domain 6 in RoBINS‐I (bias in measurement outcomes) and domain 4 in RoB2 (bias in measurement of the outcome). Accordingly, the large majority of studies (30/31) were deemed to be at low risk of bias for the measurement of VPT. However, overall, there was a high risk of bias among both the non‐RCTs and RCTs for their own primary outcomes, due to significant uncontrolled confounders. 88% of the non‐RCTs (22/25) were ranked as being at serious risk of bias using RoBINS‐I (Table 1). In addition, 67% of the RCTs (4/6) were judged as being at high risk of bias in the RoB2 tool (Table 2).

**TABLE 1:** Quality assessment of non‐randomised controlled trials using ROBINS‐I tool (risk of bias in non‐randomised studies of interventions).

	Risk of bias domains							Overall
	D1	D2	D3	D4	D5	D6	D7	
Krøigård et al. (2021)	x	−	+	+	x	+	+	x
van Gerven et al. (1994)	x	+	+	−	x	+	+	x
Hilkens et al. (1995)	x	+	+	+	x	+	+	x
Hilkens et al. (1997)	x	x	+	+	x	+	x	x
Hilkens et al. (1996)	x	x	+	+	x	+	x	x
Openshaw et al. (2005)	x	+	x	−	+	+	+	x
Hammond et al. (2019)	x	x	+	−	x	+	−	x
Goel et al. (2008)	x	+	+	+	!	+	x	!
Kokotis et al. (2016)	x	+	+	+	x	−	+	x
van den Bent et al. (2002)	x	+	+	+	−	+	x	x
Postma et al. (1999)	x	x	−	−	x	+	x	x
Verstappen et al. (2003)	x	x	−	−	x	+	x	x
Moore et al. (2003)	x	+	+	+	x	+	x	x
Von Schlippe et al. (2001)	x	−	+	+	x	+	−	x
Krøigård et al. (2021)	x	−	+	+	x	+	x	x
Nielsen et al. (2022)	−	+	+	+	+	+	+	−
Pronk et al. (1998)	x	x	+	−	x	+	x	x
Postma et al. (1999)	x	−	+	x	x	+	x	x
Elderson et al. (1989)	x	x	!	−	x	+	x	!
Marstrand et al. (2021)	x	−	x	+	+	+	x	x
Ferdousi et al. (2015)	x	+	+	+	x	+	+	x
Hovestadt et al. (1992)	x	−	x	−	x	+	−	x
Szpejewska et al. (2022)	x	x	+	+	x	+	−	x
Hershman et al. (2011)	x	x	+	−	x	+	x	x
Attal et al. (2009)	x	+	+	+	x	+	x	x

*Note:* D1 – bias due to confounding; D2—bias due to selection of participants; D3—bias in classification of interventions; D4—bias due to deviations from intended interventions; D5—bias due to missing data; D6—bias in measurement outcomes; D7—bias in selection of reported results; (+) low risk; (−) moderate risk; (x) serious risk; (!) critical.

**TABLE 2:** Quality assessment of randomised controlled trials using ROB2 tool (Risk of Bias 2).

	D1	D2	D3	D4	D5	Overall
Roberts et al. (1997)	+	−	x	+	+	x
Hammond et al. (2019)	+	−	+	+	+	−
Davis et al. (2005)	+	+	+	+	+	+
Planting et al. (1999)	−	x	x	+	x	x
Hilpert et al. (2005)	−	−	x	+	x	x
van der Hoop et al. (1990)	+	x	x	+	+	x

*Note:* D1—bias arising from the randomization process; D2—bias due to deviation from intended interventions; D3—bias due to missing outcome data; D4—bias in measurement of the outcome; D5—bias in detection of the reported result; (+) low risk; (−) some concerns; (x) high risk.

### 3.1 Change in VPT in Chemotherapy‐Treated Patients

**FIGURE 2:**
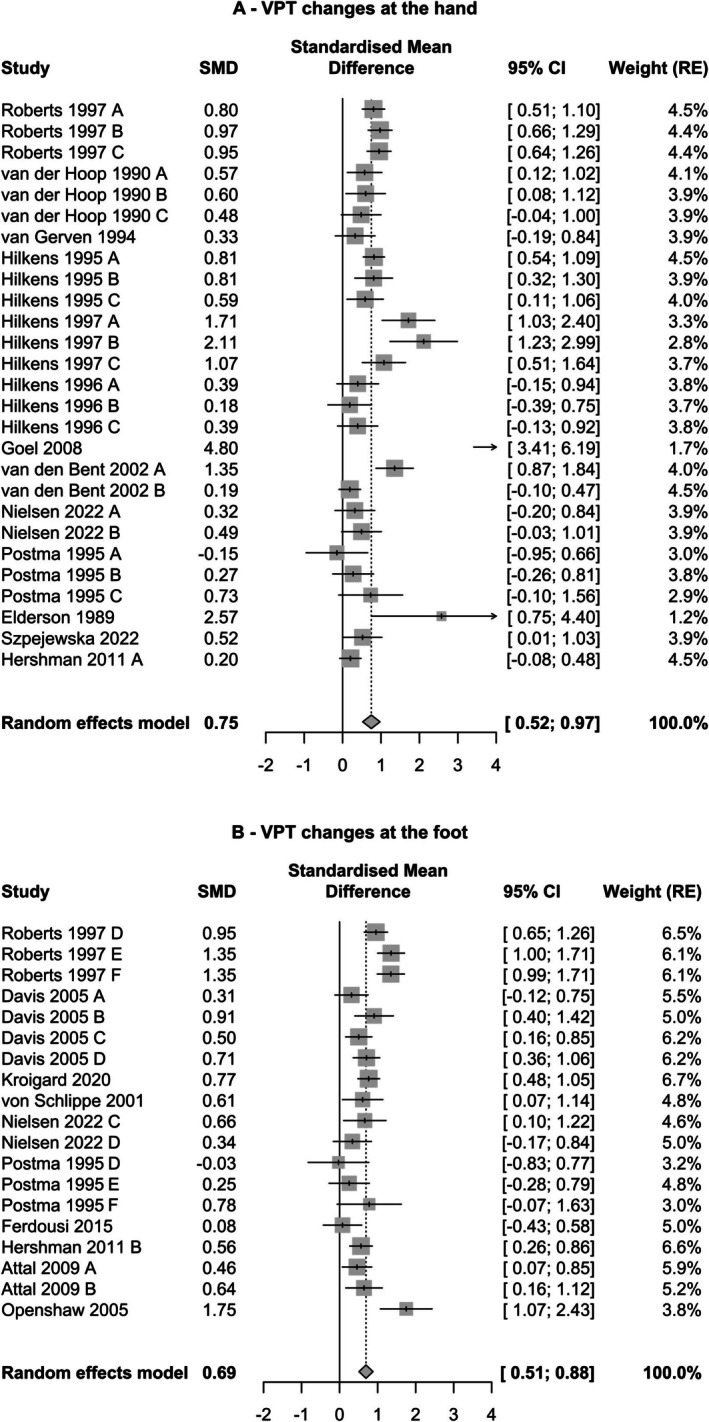
Forest plots for the change in vibration perception thresholds (VPT) following chemotherapy measured (A) in the hand and (B) at the foot. Note the small Goel et al. (2008) study in A is an outlier and its standardised mean difference is off the scale.

Random effects meta‐analyses were conducted separately for VPT measurements in patients' hands and feet. The estimated pooled standardised mean differences (SMDs) indicated moderate to large effects of chemotherapy agents on VPT from baseline in patients with cancer, in both the hand (Figure 2A) and foot (Figure 2B). In the primary random‐effects meta‐analysis of VPT changes in the hand, the pooled SMD was 0.75, 95% CI [0.52, 0.97], indicating a moderate to large loss of sensitivity to vibration following chemotherapy compared against baseline. There was substantial between‐study heterogeneity (*τ* ^2^ = 0.27; *I* ^2^ = 77.10%; *H* = 2.09). For the foot measurements of VPT, the pooled SMD was 0.69, 95% CI [0.50, 0.88], which is consistent with a moderate to large deterioration in sensitivity after chemotherapy. Heterogeneity was similar as for hand studies (*τ* ^2^ = 0.11; *I* ^2^ = 69.10%; *H* = 1.80). Statistical comparison using an independent *z*‐test showed the difference between the pooled SMD for the hand and foot was not significant (*Z* = 0.36, *p* = 0.722). The increase in VPT at both hands and feet indicates that patients are less able to detect vibration following chemotherapy, consistent with the development of impaired Aβ nerve function.

**FIGURE 3:**
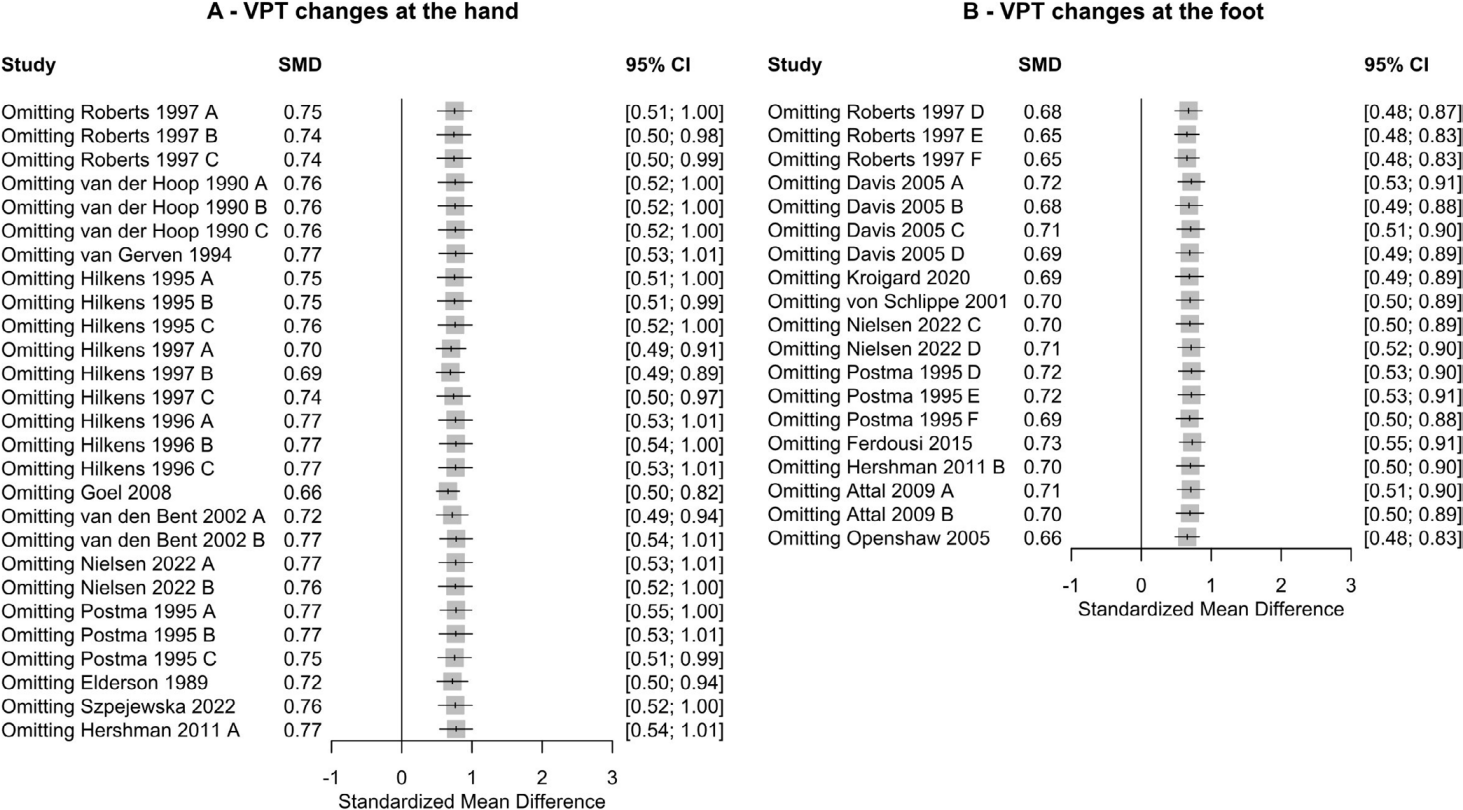
Leave one out plots for vibration perception threshold (VPT) change following chemotherapy. (A) in the hand; (B) at the foot.

The robustness of these findings was evaluated with leave‐one‐out analyses. Serial sequential omission of each study had minimal impact on the pooled effects for either hand or foot measurements (Figure 3A,B, respectively). No single study changed the point estimate or confidence interval enough to affect the overall conclusions, and none of the leave‐one‐out models indicated a statistically significant difference relative to the full model. This indicates that the observed deterioration in vibration sensitivity in the hands and feet is not driven by any one study.

**FIGURE 4:**
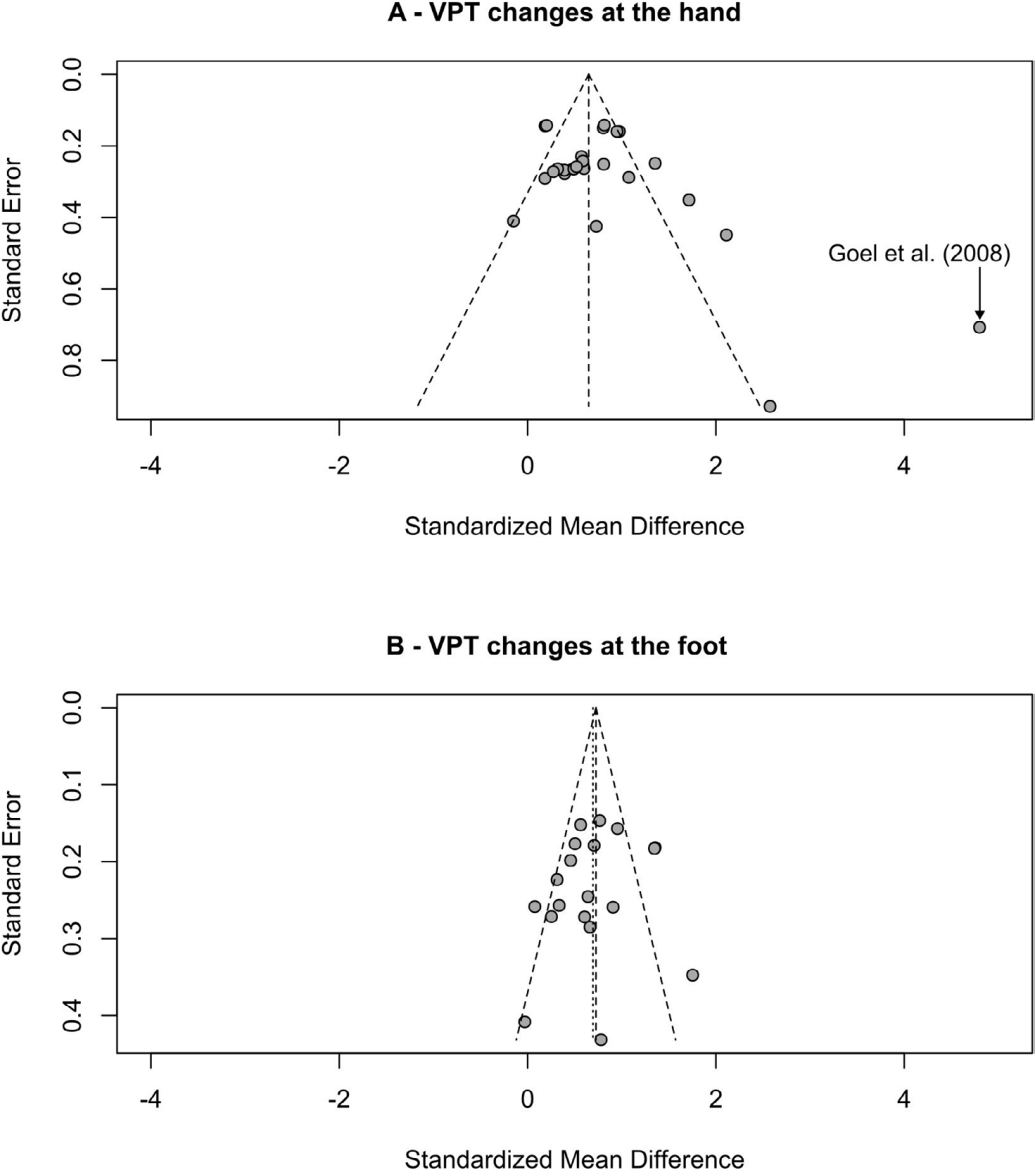
Funnel plots for vibration perception threshold (VPT) change following chemotherapy. (A) in the hand; (B) at the foot.

Visual inspection of funnel plots (Figure 4) reveals one outlier study with a larger effect than the pooled estimate when VPT is measured at the hand. However, neither Egger's test (*t*(25) = 1.92, *p* = 0.066) nor the Begg‐Mazumdar test (*Z* = 0.27, *p* = 0.786) finds significant asymmetry. We conducted a sensitivity analysis by excluding the study in question which resulted in a pooled SMD = 0.66, 95% CI [0.50, 0.82]. Therefore, the moderate to high effect of chemotherapy on VPT remains robust, with reduced heterogeneity (*τ* ^2^ = 0.11; *I* ^2^ = 68.40%; *H* = 1.78) and no significant bias per Egger's (*p* = 0.312) and the Begg‐Mazumdar tests (*p* = 0.808).

Neither Egger's test (*t*(17) = −0.86, *p* = 0.403) nor the Begg‐Mazumdar (*Z* = −0.80, *p* = 0.421) shows significant asymmetry of the funnel plot when VPT is measured at the feet.

Several studies reporting on the change in VPT from baseline in chemotherapy patients could not be included in the meta‐analysis as they did not provide mean VPT readings with error values in patients at baseline and following chemotherapy. Planting et al. (1999) reported an increase in the median values of VPT 3 months following Cisplatin treatment compared to baseline at the hand. Pronk et al. (1998) showed increased VPT in patients treated with Docetaxel with and without corticosteroid co‐medication (50% and 20% increase respectively). These findings are all consistent with the meta‐analytic results indicating that there are chemotherapy‐associated increases in VPT.

Considering that different measurement devices set at different vibration frequencies were used across studies (likely contributing substantially to the heterogeneity between studies), it is difficult to determine what the standardised pooled effect translates to in terms of change in physical stimulus required for detection of vibration following chemotherapy. However, we have identified 11 cohorts within reviewed studies which were all conducted using the Vibrameter (Somedic SenseLab AB, Stockholm, Sweden) at a vibration frequency of 100 Hz with measurements done at the hands. These studies include 221 participants with a weighted average baseline VPT of 0.76 μm, and a post chemotherapy VPT of 2.56 μm corresponding to a 3‐fold rise. This translates to a pooled SMD of 0.58, 95% CI [0.43, 0.73] with very low heterogeneity (*τ* ^2^ = 0.005; *I* ^2^ = 7.28%; *H* = 1.04).

### 3.2 Exploratory Meta‐Analysis of Changes in VPT by Mid‐Treatment

Most studies included more than pre‐ and post‐ measurements of VPT, offering the opportunity to estimate the effect of chemotherapy on VPT earlier in treatment and assess whether early detection of neuropathic changes is plausible. We performed an exploratory analysis to address this question. However, the timing and frequency of intermediate measurements varied significantly across studies. For example, Roberts et al. (1997) reported VPT measurements at six time points during the chemotherapy treatment, while Davis et al. (2005) conducted only one mid‐treatment measurement in addition to baseline and post‐treatment measurements. To enable a consistent comparison, we conducted an analysis of baseline versus mid‐treatment VPT measurements. Where mid‐treatment measurements were not available, the closest measurement to mid‐treatment was extracted. If measurements before and after were equidistant from mid‐treatment, the later measurement was selected.

A total of 15 studies of VPT measured at the hands (total *N* = 349) and 15 studies of VPT measured at the feet (total *N* = 457) were eligible for this exploratory analysis. The results revealed significant pooled effects both at the hand (SMD = 0.58, 95% CI [0.09, 1.07]) and the foot (SMD = 0.38, 95% CI [0.28, 0.48]). The heterogeneity of VPT measurements at the hand was considerable (*τ* ^2^ = 0.80; *I* ^2^ = 78.50%; *H* = 2.15) with one highly influential study (Goel et al. 2008). Leaving the study out of the analysis results in a much reduced, but still significant, pooled effect (SMD = 0.31, 95% [0.15, 0.47]) more in line with the effect at the feet (which had markedly lower heterogeneity; *τ* ^2^ < 0.01; *I* ^2^ = 15.60%; *H* = 1.09). Forest and funnel plots as well as leave‐one‐out analyses can be found in (Figures [Link], [Link]).

The findings reveal smaller effects mid‐treatment than at the end of treatment. This likely reflects the cumulative nature of neurotoxicity, particularly for platinum‐based agents, where increasing cumulative dose is associated with more severe neuropathy (Burgess et al. 2021; Zhou et al. 2026).

### 3.3 Association Between Chemotherapy Agent and Dose With Changes in VPT

A random‐effects meta‐regression was performed to examine if changes in VPT measured in the hand differed by chemotherapy type. Platinum‐based agents (Cisplatin, Carboplatin, Oxaliplatin) were used as the reference category and compared with taxane‐based agents (Paclitaxel, Docetaxel) and combination regimens including both platinum and taxanes (Table 3).

**TABLE 3:** Random‐effects meta‐regression of standardised mean differences (SMD) in hand vibration perception thresholds by chemotherapy type.

Chemotherapy	*N*	SMD	95% CI		*p*	SMD vs. ref	95% CI		*p*
Platinum (ref)	14	0.68	0.50	0.86	< 0.001				
Taxane	8	0.34	0.06	0.62	0.018	−0.34	−0.68	−0.01	0.047
Combination	4	1.21	0.79	1.63	< 0.001	0.53	0.07	0.99	0.023

*Note:* Chemotherapy type moderation test *χ* ^2^ (df = 2) = 11.66, *p* = 0.003.

Platinum‐based chemotherapy, taxane‐based therapy, and combination therapy were all associated with a significant increase in VPT. For platinum‐based chemotherapy this corresponded to a moderate worsening of vibration perception following treatment. The change was relatively smaller in studies in which patients were treated exclusively with taxanes and larger in studies where treatment was a combination of a platinum and taxane. Relative to platins as reference, both the decrease of effect for taxanes and the increase of effect in combined treatments reached statistical significance. This suggests potentially biologically meaningful patterns whereby taxanes impact vibration less than platinum agents, whereas combined regimens exert an additive effect on vibration perception loss. The chemotherapy agent explained approximately 32.71% of between‐study heterogeneity, indicating that the class of chemotherapy agent is an important contributor to the observed variance across studies.

The same meta‐regression for studies measuring VPT at the feet did not reveal significant moderation by chemotherapy type on change in VPT (Table 4).

**TABLE 4:** Random‐effects meta‐regression of standardised mean differences (SMD) in foot vibration perception thresholds by chemotherapy type.

Chemotherapy	*N*	SMD	95% CI		*p*	SMD vs. ref	95% CI		*p*
Platinum (ref)	9	0.79	0.53	1.05	< 0.001				
Taxane	5	0.65	0.24	1.05	0.002	−0.14	−0.62	0.34	0.565
Combination	5	0.55	0.20	0.91	0.002	−0.24	−0.68	0.21	0.293

*Note:* Chemotherapy type moderation test *χ*2(df = 2) = 0.72, *p* = 0.699.

Several studies reported a dose‐dependent effect of chemotherapy on VPT. Pronk et al. (1998) and Postma et al. (1999) both observed increasing VPT with higher cumulative doses of Docetaxel and Paclitaxel. Postma et al. (1999), who compared three Paclitaxel dose groups administered every 3 weeks, found that the highest dose group (250–300 mg/m^2^) showed a 0.89 greater standardised mean change in VPT compared with the lowest dose (135 mg/m^2^; *p* = 0.03). van den Bent et al. (2002) demonstrated that cumulative Cisplatin dose and per‐cycle dose intensity (70 mg/m^2^ vs. 50 mg/m^2^) were independent predictors of post‐treatment VPT.

Elderson et al. (1989) reported that clinical signs and symptoms of neuropathy, detected through routine neurological examination, typically appeared after cumulative Cisplatin doses of around 450 mg/m^2^ (after approximately 18 weeks of treatment with 75 mg/m^2^ administered every 3 weeks). They noted that increases in VPT were detectable 6–8 weeks earlier, suggesting that VPT may serve as an early indicator of subclinical neurotoxicity.

### 3.4 Relationship Between VPT and Patient Reported Outcomes/Neurological Examination

Several studies investigated associations between VPT and patient‐reported outcomes, symptoms, or neurological examination findings, with mixed findings. Krøigård et al. (2021) found that changes in VPT following treatment with Oxaliplatin correlated significantly with the Inflammatory Neuropathy Cause and Treatments—Sensory Sum Score (INCAR‐SS), but not with the Neuropathy Symptom Score (NSS).

Similarly, Kokotis et al. (2016) reported that change in VPT correlated positively with Neuropathy Impairment Score (NIS) values, while Hovestadt et al. (1992) found a high correlation (*r* = 0.81) between the change in VPT and patient‐reported signs and symptom sum scores for patients treated with Cisplatin. Hershman et al. (2011) found longitudinal associations between VPT changes and symptoms recorded on the Functional Assessment of Cancer Therapy—Taxane (FACT‐Tax) neurotoxicity component, including hand numbness, foot numbness, hand pain, and overall neurotoxicity burden. These findings support that changes in VPT correspond with some indicators of CIPN, such as higher symptom burden or worse clinical grading of neuropathy.

In contrast, van der Hoop et al. (1990) reported mixed findings. Correlations between VPT and symptom scores after four and six cycles of Cisplatin were not significant (*r = 0*.32 and *r =* 0.35 respectively). However, correlations between VPT and sign scores were significant both after four and six chemotherapy cycles (*r* = 0.63 and *r* = 0.54 respectively). Similarly, Szpejewska et al. (2022) found only weak correlations between VPT and NCI CTC scores (*r* = 0.25). They noted that VPT increased later than NCI CTC‐reported neuropathy and returned to baseline during follow‐up.

### 3.5 Change in VPT in Patients With CIPN

Only a small number of studies reported VPT outcomes stratified by whether or not patients developed CIPN (*n* = 5). The available data were heterogeneous in design, reporting, and in how CIPN was defined. Goel et al. (2008) observed significantly higher VPT in patients treated with chemotherapy that developed moderate‐to‐severe neuropathy (grade 2–3) compared with those with none or mild neuropathy (grade 0–1), as measured by the National Cancer Institute Common Toxicity Criteria (NCI CTC). Krøigård et al. (2021) reported significant differences in VPT assessed with the calibrated tuning fork between patients who developed neuropathy (rTNS > 5) and those who did not following Oxaliplatin treatment. However, the difference was present prior to chemotherapy treatment and did not increase at any point following start of treatment. Marstrand et al. (2021) reported significantly higher VPT in breast cancer patients (90% of whom were treated with taxanes) with CIPN compared with healthy controls, but found no difference in VPT between cancer patients with and without CIPN. Participants were allocated to the CIPN group if they reported any one of the following: pain, tingling, numbness, burning, shooting feelings in hands or feet or trouble fastening buttons. They suggested that the elevated VPT might relate to breast cancer itself rather than a complication of the chemotherapy. Ferdousi et al. (2015), studying oesophageal cancer patients receiving Oxaliplatin or Cisplatin, found no significant difference in VPT between those who did and did not develop grade 1 paraesthesia using the NCI CTC criteria. Hammond et al. (2019) similarly found no significant difference in median VPT 6 months after Docetaxel therapy between patients with and without CIPN. Patients were stratified based on the self‐report version of the Leeds Assessment of Neuropathic Symptoms and Signs pain score (S‐LANSS score < 12 vs. ≥ 12).

Only two studies (Ferdousi et al. 2015; Goel et al. 2008) provided mean change in VPT values with corresponding error estimates, but the remaining studies used inconsistent outcome definitions (median/IQR data only) or post‐treatment measures without baseline, so a meta‐analysis of change in VPT in CIPN versus non‐CIPN groups was not feasible.

## 4 Discussion

This systematic review and meta‐analysis demonstrate that vibration perception threshold (VPT) consistently increases following chemotherapy (reduced sensitivity), with moderate pooled effect sizes observed at both hands and feet. For the hands specifically this corresponds to around a 3‐fold increase in the amplitude of vibration stimulus required before it can be detected—consistent with a substantial deterioration in sensory function. These findings add to the evidence that suggests that chemotherapy impairs sensory nerve function, including of large fibres. VPT therefore is a promising objective measure that can contribute to the assessment of peripheral neurotoxicity (Elliott et al. 2009). An exploratory analysis demonstrated that smaller but robust effects of chemotherapy on VPT can be detected earlier during treatment which is promising given that the aim is to detect signs of neuropathy before they become severe and chronic. We also found evidence of chemotherapy type moderating the change in VPT. Combination treatments with both platinum‐ and taxane‐based agents produced the largest impact on VPT and treatments exclusively consisting of taxane‐based agents related to the smallest deterioration of sensitivity to vibration. However, this moderation was only significant when VPT was measured at the hands but not the feet. These results at the hand are in line with previous studies showing that administering more than one neurotoxic agent increases the incidence and severity of CIPN (Argyriou et al. 2012; Park et al. 2013).

The finding that differences in impact of different chemotherapy regimens can be detected at the hands but not feet may be a result of higher sensitivity of VPT measurements at the hands, indicating that testing at the hands may be more beneficial for detecting CIPN even though symptoms more often develop earlier, are more severe and persist longer at the feet (Prager et al. 2023; Wang et al. 2019; Zajączkowska et al. 2019). This agrees with findings from Dujmović et al. (2025) who show less age‐related change, lower within‐ and between‐participant variance of sensory testing (vibration detection, cold detection, and warm detection thresholds) at the thenar eminence when compared to the dorsum of the foot. For example, there was no significant deterioration in vibration detection between younger (average of 21) and older (average age of 58) participants at the thenar eminence (effect size *d* = 0.39 [−0.23, 0.99]) whereas the same groups of subjects showed a large difference at the dorsum of the foot with older subjects having poorer function (*d* = 1.22 [0.55, 1.87]). When combined with the fact that the magnitude of the pooled effect of chemotherapy on VPT was not statistically different at the hands and feet provides support for the use of the hands as a test site.

Two key issues complicate the interpretation of the results of this systematic review and meta‐analysis, namely methodological heterogeneity between studies and the inconsistent definition of CIPN (or indeed its complete absence). Our meta‐analysis showed a consistent worsening of vibration perception following chemotherapy treatment. However, individual studies varied significantly, with heterogeneity in methodologies including cancer type, prior treatments, cumulative chemotherapy dose, regimen intensity, and duration (details in Table S1). These differences likely contributed to between‐study variability despite the consistent overall direction of effect. However, we found there was a relatively low risk of bias in VPT measurements in the studies, although they were, more generally, of low quality.

Although there was variation in measurement equipment employed across studies, they all measure the same psychophysical construct and are widely used in research. The large majority of these studies use haptic actuator‐based vibration devices allowing higher measurement precision and sensitivity to detect changes than the standard calibrated tuning fork, as noted in several recent studies (e.g., see Dujmović et al. 2025; Temlett 2009). This suggests that, despite some individual device variability, the consistent increase in VPT across devices supports the robustness of the observed chemotherapy‐related deterioration in vibration sensitivity. Study heterogeneity poses issues in estimating the potential of VPT for early detection of CIPN and precludes fine‐grained meta‐regression analysis into correlates and confounding factors. The heterogeneity also makes the estimate of pooled effects less precise with confidence intervals ranging from medium‐sized (SMD = 0.52) to quite a large effect (SMD = 0.97).

The second major challenge in this field is the lack of a gold standard diagnostic criterion for CIPN. Most studies did not clearly distinguish participants with and without CIPN, and those that did relied on inconsistent criteria or the presence of nonspecific symptoms such as pain, which affects only approximately 30% to 40% of patients with CIPN (D'Souza et al. 2025; Smith et al. 2011; Velasco et al. 2017). Many patients with CIPN may experience troublesome symptoms such as numbness, paraesthesia, or motor dysfunction without pain. Furthermore, patients may experience pain for reasons other than the chemotherapy, including pain directly associated with their underlying cancer (Laycock et al. 2025). This can lead to patients being allocated to CIPN groups based on pain not related to CIPN or to non‐CIPN groups due to lack of pain even though they developed CIPN. The inadequate group allocation makes it less likely to detect differences between the groups. For example, several studies found no significant difference in VPT between those “with” and “without” CIPN when classification relied on individual symptoms such as pain (Ferdousi et al. 2015; Hammond et al. 2019). Overall, the small number of studies, the variability in CIPN definitions, and the inconsistent approaches to measuring VPT all limit the strength of any pooled conclusions. The lack of standardised classification of CIPN also limits meaningful evaluation of VPT as a biomarker, precluding the ability to define diagnostic thresholds, sensitivity, specificity, and minimal clinically important differences. The current evidence does not establish a consistent relationship between VPT changes and the presence of CIPN, highlighting a gap in the literature to answer this clinically important question.

A further challenge to making overarching conclusions about the impact of neurotoxic chemotherapy on VPT comes from the lack of research into chemotherapy agents outside the platinum and taxane families of drugs. Only one such study fulfilled inclusion criteria for this review. Therefore, there is a gap in knowledge about other neurotoxic chemotherapy agents such as vinca alkaloids, proteasome inhibitors (such as bortezomib), and also anti‐angiogenesis medication (such as thalidomide). This is an important area to research as different chemotherapy agents rely on different mechanisms of action and different mechanisms of inducing peripheral neuropathy (Zajączkowska et al. 2019). Different classes of chemotherapy can cause different prominent symptoms as part of CIPN, which may impact how reliably VPT changes (Staff et al. 2017). However, it is important to note that platinum and taxane agents are so prominent in research because of the combination of high neurotoxicity and frequency of use for treatment of very common cancers such as colorectal and breast cancers. Accordingly, our results provide strong evidence of the utility of VPT measurement in CIPN research and potentially diagnosis when platinum and taxane treatments are prescribed.

Most studies' exclusion criteria aim to control for a variety of confounding factors (e.g., diabetes, other neuropathies, other pain conditions, malnutrition, previous exposure to neurotoxic agents) meaning that a meta‐regression to estimate the impact of these factors is not possible. Studies which have wider inclusion criteria, on the other hand, do not track confounding factors or have not made the data available for accumulation and analysis. It is therefore unknown whether these factors influence the changes in VPT. This is an important area for further research, as many of these factors increase the risk of developing CIPN. For example, people with diabetes mellitus have been shown to have 60% higher risk of developing CIPN (Gu et al. 2021).

Furthermore, cancer itself, either through direct effects or cancer‐associated paraneoplastic antibodies, may cause neuropathy and subsequent changes to VPT over time independent of chemotherapy exposure. This represented an intrinsic limitation of within‐subject designs (Chiu et al. 2023; Lipton et al. 1987; Zoccarato et al. 2021). The control group for drawing inferences about those contributions would need to be a cohort with the same diagnosis not treated with neurotoxic chemotherapy agents. Marstrand et al. (2021) was the only study included to incorporate such a comparison, examining breast cancer patients treated with chemotherapy and those managed without chemotherapy, and reported no significant difference in VPT between groups. However, the absence of pre‐treatment baseline VPT measurements limits the confidence in this interpretation and consequently was not eligible for inclusion in our meta‐analysis.

Apart from better differentiation between CIPN and non‐CIPN patients, and research into other neurotoxic agents, our review also revealed that most studies have small sample sizes, poor data accessibility, and a limited number of measurements during chemotherapy treatment. We also note that none of the reviewed papers provide data in line with FAIR principles (Wilkinson et al. 2016). This meant data was extracted from information published in the papers. Further, even though papers routinely state data is available upon request, none of our requests yielded data. If VPT measurements and sensory testing in general are to be useful both in research and clinical practice, there is also a need for more frequent testing with larger sample sizes. The time burden, equipment costs, and the need for training to administer sensory testing create substantial obstacles towards these goals. Current work on developing reliable, self‐administered sensory testing, including VPT measurement, may provide the advances needed to undertake key research into early detection of CIPN via continual monitoring of sensory function (Dujmović et al. 2025).

## 5 Conclusion

To our knowledge this is the first systematic review with a meta‐analysis of a QST measure for CIPN—demonstrating the feasibility and utility of such an evidence synthesis approach. We find that neurotoxic chemotherapy, particularly with platinum and taxane agents, is consistently associated with a moderate reduction in vibration sensitivity, reflecting large‐fibre sensory impairment. The effect is similar across the upper and lower limbs, suggesting both hands and feet are valid testing sites for future studies. The lack of standardised CIPN diagnostic criteria limits conclusions regarding whether VPT can be used as an effective biomarker for the early identification of CIPN. Future longitudinal studies incorporating consistent CIPN definitions and diverse chemotherapy classes are needed to clarify the role of VPT as a biomarker for early detection and monitoring of chemotherapy‐induced peripheral neuropathy.

## Author Contributions

F.K. conducted the review and wrote the first draft of the paper. F.K., M.H., M.D. reviewed the titles and abstracts and extracted the data. F.K. and M.H. completed the risk of bias assessments. F.K. and M.D. completed the meta‐analysis. J.P.D., T.P. and M.D. conceptualised the study. All the authors reviewed the drafts of the paper, and all approved the final submitted version.

## Funding

The authors have nothing to report.

## Disclosure

The authors have nothing to report.

## Supporting information


**Table S1:** Characteristics of studies included in the review.

**Figure S1:** Forest plots for the change in vibration perception thresholds (VPT) from baseline to mid‐chemotherapy timepoint measured A—in the hand and B—at the foot. Note the small Goel et al. (2008) study in A is an outliersand its standardised mean difference is off the scale.

**Figure S2:** Funnel plots for vibration perception threshold (VPT) change from baseline to mid‐chemotherapy timepoint. A—in the hand; B—at the foot.

**Figure S3:** Leave one out plots for vibration perception threshold (VPT) change from baseline to mid‐chemotherapy timepoint. A—in the hand; B—at the foot.

**Methods S1** Full search strategy.
