# Supplementary material for: Utility of Vibration Perception Thresholds as a Biomarker of Chemotherapy‐Induced Peripheral Neuropathy: A Systematic Review and Meta‐Analysis
Source: Eur J Pain. 2026 Jul 1;30(6):e70319. doi: 10.1002/ejp.70319 (PMC13324228; doi:10.1002/ejp.70319)
Supplement: Supplementary file 1 — Table S1: Characteristics of studies included in the review. [file EJP-30-0-s004.docx]

| **Table S1**  *Characteristics of studies included in the review* | | | | | | | | | |
| --- | --- | --- | --- | --- | --- | --- | --- | --- | --- |
| Paper | Cancer type (most common) | Vibration Testing Method | Location | Neurotoxic Agent | Group | Number of Participants | Age | | %Female |
|  |  |  |  |  |  |  | mean (SD) | median (range) |  |
| Hammond (2020) | Breast | TSAII VSA3000 | Hand | Docetaxel | Control | 26 | 53.0 (10.3) |  | 100 |
|  | Breast | TSAII VSA3000 | Hand | Docetaxel | Physical Therapy | 22 | 56.3 (9.9) |  | 100 |
| Davis (2005) | Non-small-cell lung cancer | Vibratron II | Foot | Paclitaxel and Carboplatin | NA | 117 |  | 58 (22-78) | 45.3 |
| Hilpert (2005) | Ovarian | Vibrameter | Hand and Foot | Paclitaxel and Carboplatin |  | 71 |  |  | 100 |
| Planting (1999) | Head and Neck | Vibrameter type IV | Hand | Cisplatin | Placebo | 37 |  | 54 (36-67)` | 24.3 |
|  | Head and Neck | Vibrameter type IV | Hand | Cisplatin | Amifostine | 37 |  | 54 (35 - 69) | 27.1 |
| Roberts (1997) | Ovarian | Vibratron II | Hand and Foot | Cisplatin | NA | 196 | 54.8 |  | 100 |
| van der Hoop (1990) | Ovarian | Vibrameter type III | Hand | Cisplatin | Placebo | 22 |  | 53 (39-65) | 100 |
|  | Ovarian | Vibrameter type III | Hand | Cisplatin | Org 2766 0.25mg/m2 | 17 |  | 52 (31-65) | 100 |
|  | Ovarian | Vibrameter type III | Hand | Cisplatin | Org 2766 1mg/m2 | 16 |  | 53 (22-66) | 100 |
| Nielsen (2022) | Colorectal | VibroSense Meter II | Hand and Foot | Oxaliplatin | Oxaliplatin | 15 |  | 57 (42-72) | 67 |
|  | Ovarian | VibroSense Meter II | Hand and Foot | Paclitaxel and Carboplatin | Paclitaxel | 16 |  | 60 (47-77) | 100 |
| Szpejewska (2022) | Colon | Vibrameter | Hand | Oxaliplatin |  | 17 |  | 67 (36 - 72) | 53 |
| Kroigard (2021) | Colorectal / Oesophageal | Biothesiometer | Foot | Oxaliplatin |  | 69 |  | 65 (37-38) | 29 |
| Marstrand (2021) | Breast | VibroSense Meter | Hand | Docetaxel or Paclitaxel | Breast Cancer + Chemo | 30 |  | 56 (35 - 77) | 100 |
|  | Breast | VibroSense Meter | Hand | NA | Breast Cancer + No Chemo | 26 |  | 68 (53 - 83) | 100 |
|  | NA (Healthy Controls) | VibroSense Meter | Hand | NA | Controls | 22 |  | 64 (42 - 80) | 100 |
| Kroigard (2020) | Gastrointestinal | Biothesiometer | Foot | Oxaliplatin | Oxaliplatin | 60 |  | 66 (37 - 78) | 30 |
|  | NA (Healthy Controls) |  | Foot | NA | Controls | 46 |  | 51 (40 - 80) | 100 |
| Hammond (2019) | Breast | TSAII VSA3000 | Hand | Docetaxel |  | 48 | 61.5 (23.33) |  | 100 |
| Kokotis (2016) | Colorectal | Graduated Tuning Fork | Foot | Oxaliplatin |  | 31 | 65.0 (8.76) |  | 39 |
| Ferdousi (2015) | Oesophageal | Neuroesthesiometer | Foot | Oxaliplatin and Cisplatin |  | 13 | 63.8 (10) |  | 5 |
| Hershman (2011) | Breast | Biothesiometer | Hand and Foot | Paclitaxel |  | 50 |  | 48 (28-78) | 100 |
| Attal (2009) | Colorectal | Vibrameter | Foot | Oxaliplatin |  | 28 | 58.9 (11.5) |  | 43 |
|  | Pancreatic | Vibrameter | Foot | Cisplatin |  | 20 | 57.0 (12.2) |  | 15 |
| Goel (2008) | Ovarian | Vibratron II | Hand | Ixabepilone |  | 44 |  | 57 (30-81) | 88.6 |
| Openshaw (2005) | Breast | Case IV System | Foot | Paclitaxel |  | 21 |  | 42 (29-62) | 100 |
| Moore (2003) | Gynaecological | Vibratron II | Hand and Foot | Cisplatin or Paclitaxel |  | 27 |  | 61 (32 -83) | 100 |
| Verstappen (2003) | Ovarian | Vibrameter type IV | Hand and Foot | Paclitaxel |  | 18 |  | 57.5 (40-70) | 100 |
| van den Bent (2002) | Ovarian | Vibrameter type IV | Hand | Cisplatin | Carboplatin pretreated | 31 |  | 56 (32 - 75) | 100 |
|  | Ovarian | Vibrameter type IV | Hand | Cisplatin | Cisplatin pretreated | 49 |  | 57 (26 - 78) | 100 |
| von Schlippe (2001) | Testicular | Biothesiometer | Foot | Cisplatin |  | 16 |  | 30 (14 - 50) | 0 |
| **Table S1**  *Characteristics of studies included in the review - continued* | | | | | | | | | |
| Paper | Cancer type (most common) | Vibration Testing Method | Location | Neurotoxic Agent | Group | Number of Participants | Age | | %Female |
|  |  |  |  |  |  |  | mean (SD) | median (range) |  |
| Postma (1999) | Ovarian | Vibrameter type IV | Hand and Foot | Paclitaxel and Cisplatin |  | 22 |  | 49.5 (23 - 63) | 100 |
| Pronk (1998) | Ovarian and Bladder | Vibrameter type IV | Hand | Docetaxel |  | 38 |  | 52 (28 - 73) | 39 |
|  | Breast | Vibrameter type IV | Hand | Docetaxel | Dexamethasone co-administered | 49 |  | 50 (31 - 73) | 0 |
| Hilkens (1997) | Colorectal | Vibrameter type IV | Hand | Cisplatin and Docetaxel | Chemotherapy cumulative dose | 55 |  | 53 (21 - 74) | 47 |
| Hilkens (1996) | Breast and Ovarian | Vibrameter type IV | Hand | Docetaxel | Chemotherapy cumulative dose | 41 |  | 53 (28 - 73) | 34.1 |
| Hilkens (1995) | Head and Neck | Vibrameter type III | Hand | Cisplatin | Dosing regime | 66 |  | 55 (34 - 71) | 77 |
|  | Ovarian | Vibrameter type III | Hand | Cisplatin |  | 21 |  | 58 (28 - 73) | 0 |
|  | Testicular | Vibrameter type III | Hand | Cisplatin |  | 20 |  | 29 (18 - 41) | 100 |
| Postma (1995) | Ovarian | Vibrameter type IV | Hand | Paclitaxel | Chemotherapy Regime | 6 |  | 61.5 (43 - 72) | 0 |
|  | Ovarian | Vibrameter type IV | Hand | Paclitaxel |  | 14 |  | 55.5 (33 - 69) | 0 |
|  | Breast | Vibrameter type IV | Hand | Paclitaxel |  | 7 |  | 56 (43 - 71) | 0 |
| van Gerven (1994) | Breast | Vibrameter | Hand | Paclitaxel |  | 8 |  | 51 (29 - 72) | 0 |
| Hovestadt (1992) | Ovarian | Vibrameter type III | Hand | Cisplatin |  | 25 |  |  | 0 |
| Elderson (1989) | Ovarian | Vibrameter type III | Hand | Cisplatin |  | 5 |  |  | 0 |

*Note.* Device manufacturers : TSAII Vibration Sensory Analyzer - VSA3000; Medoc, Israel; Vibratron II - Physitemp, Inc., NJ, USA; Vibrameter type III / IV - Somedic AB, Stockholm, Sweden; VibroSense Meter II - Vibrosense Dynamics AB, Malmo, Sweden; Biothesiometer - Bio-Medical Instruments Co., Ohio, USA; Neuroesthesiometer - Horwell, Scientific Laboratory Supplies, Wilford, Nottingham, UK; Case IV System - WR Medical Electronics, Minnesota, USA.
