# Supplementary figures and images for: Utility of Vibration Perception Thresholds as a Biomarker of Chemotherapy‐Induced Peripheral Neuropathy: A Systematic Review and Meta‐Analysis
Source: Eur J Pain. 2026 Jul 1;30(6):e70319. doi: 10.1002/ejp.70319 (PMC13324228; doi:10.1002/ejp.70319)

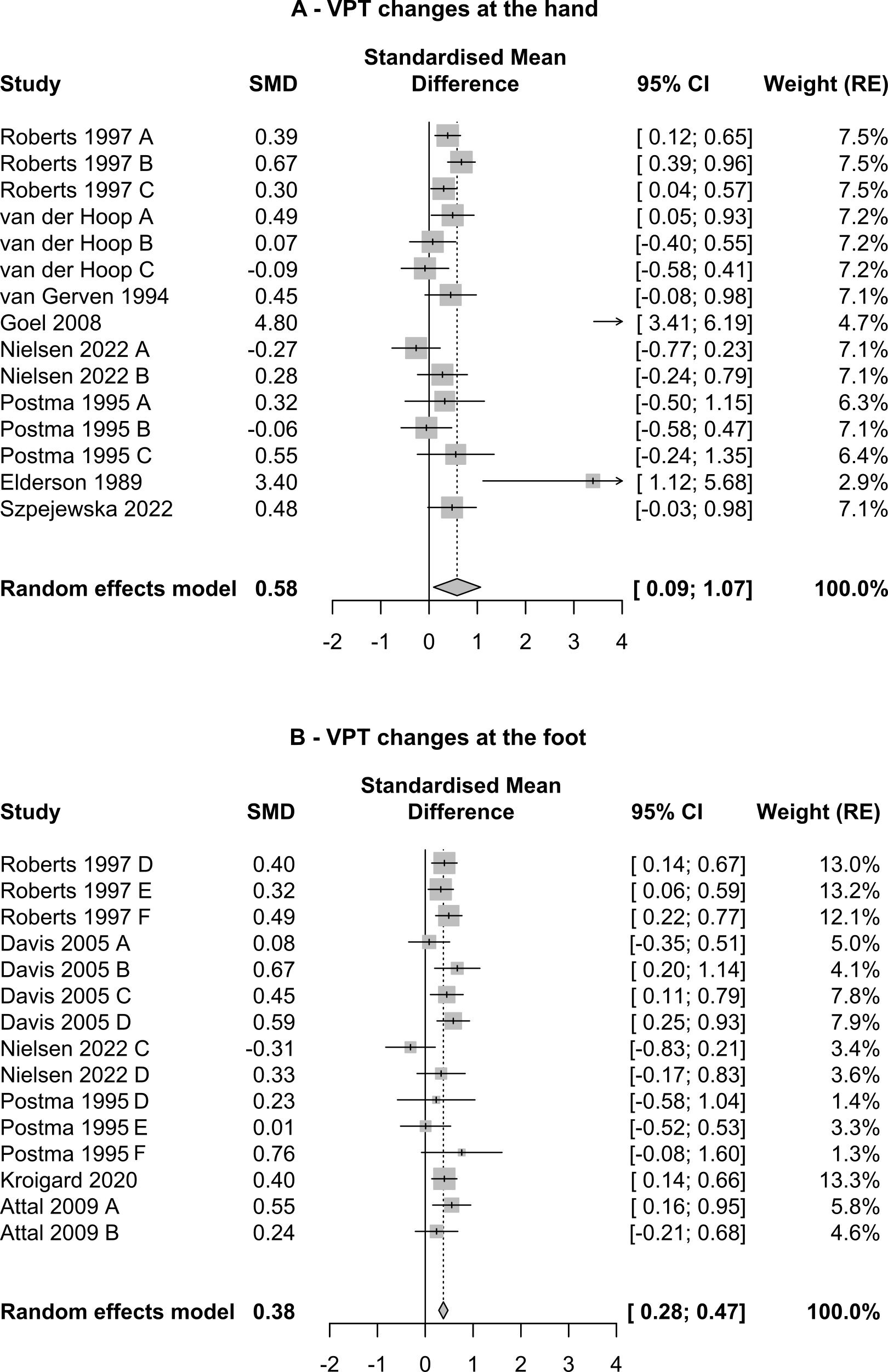

Supplement: Supplementary file 2 — Figure S1: Forest plots for the change in vibration perception thresholds (VPT) from baseline to mid‐chemotherapy timepoint measured A—in the hand and B—at the foot. Note the small Goel et al. (2008) study in A is an outliersand its standardised mean difference is off the scale. [file EJP-30-0-s003.jpg]

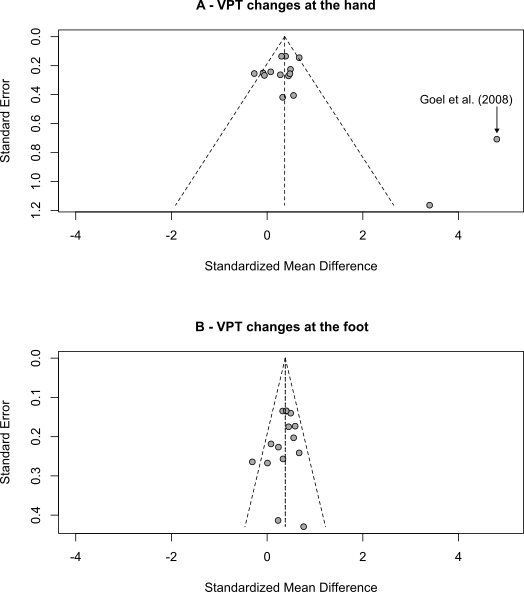

Supplement: Supplementary file 3 — Figure S2: Funnel plots for vibration perception threshold (VPT) change from baseline to mid‐chemotherapy timepoint. A—in the hand; B—at the foot. [file EJP-30-0-s001.jpg]

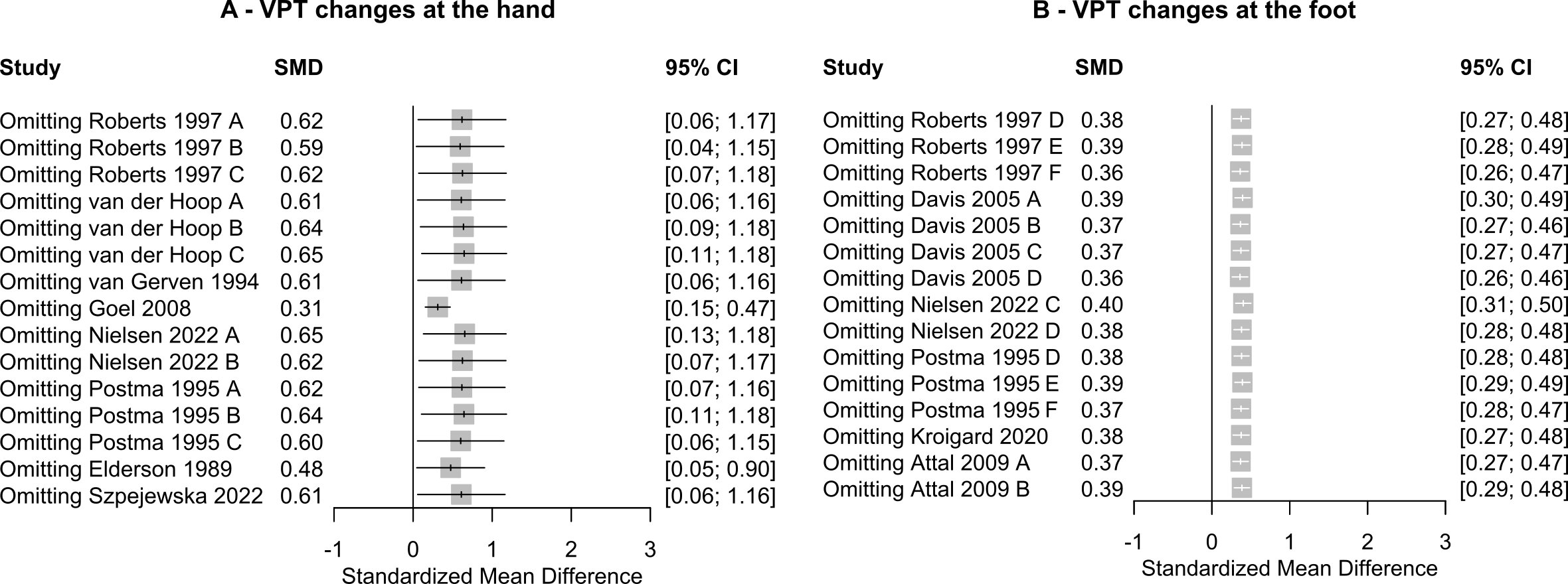

Supplement: Supplementary file 4 — Figure S3: Leave one out plots for vibration perception threshold (VPT) change from baseline to mid‐chemotherapy timepoint. A—in the hand; B—at the foot. [file EJP-30-0-s002.jpg]
