# Supplementary material for: Utility of Vibration Perception Thresholds as a Biomarker of Chemotherapy‐Induced Peripheral Neuropathy: A Systematic Review and Meta‐Analysis
Source: Eur J Pain. 2026 Jul 1;30(6):e70319. doi: 10.1002/ejp.70319 (PMC13324228; doi:10.1002/ejp.70319)
Supplement: Supplementary file 5 — Methods S1 Full search strategy. [file EJP-30-0-s005.docx]

Methods S1. Full Search Strategy

PubMed

Subject Headings

("Touch"[Mesh] OR "Vibration"[Mesh]) AND "Peripheral Nervous System Diseases"[Mesh] AND "Drug Therapy"[Mesh]

Keywords

(((peripheral neuropathy) AND (chemotherapy)) OR ((peripheral neuropathy) AND (cancer)) OR (chemotherapy induced neuropathy) OR (CIPN) OR (Chemotherapy induced peripheral neuropathy)) AND ((vibration perception) OR (vibration perception threshold) OR (VPT) OR (vibration detection) OR (vibration detection threshold) OR (VDT) OR (vibration testing) OR (vibration sensitivity))

Embase via OVID

Subject Headings

("Touch"[Mesh] OR "Vibration"[Mesh]) AND "Peripheral Neuropathy"[Mesh] AND "Drug Therapy"[Mesh]

Keywords

(((peripheral neuropathy) AND (chemotherapy)) OR ((peripheral neuropathy) AND (cancer)) OR (chemotherapy induced neuropathy) OR (CIPN) OR (Chemotherapy induced peripheral neuropathy)) AND ((vibration perception) OR (vibration perception threshold) OR (VPT) OR (vibration detection) OR (vibration detection threshold) OR (VDT) OR (vibration testing) OR (vibration sensitivity))

Cochrane Library

Subject Headings

("Touch"[Mesh] OR "Vibration"[Mesh]) AND "Peripheral Nervous System Diseases"[Mesh] AND "Drug Therapy"[Mesh]

Keywords

(((peripheral neuropathy) AND (chemotherapy)) OR ((peripheral neuropathy) AND (cancer)) OR (chemotherapy induced neuropathy) OR (CIPN) OR (Chemotherapy induced peripheral neuropathy)) AND ((vibration perception) OR (vibration perception threshold) OR (VPT) OR (vibration detection) OR (vibration detection threshold) OR (VDT) OR (vibration testing) OR (vibration sensitivity))

Web of Science

Keywords

(((peripheral neuropathy) AND (chemotherapy)) OR ((peripheral neuropathy) AND (cancer)) OR (chemotherapy induced neuropathy) OR (CIPN) OR (Chemotherapy induced peripheral neuropathy)) AND ((vibration perception) OR (vibration perception threshold) OR (VPT) OR (vibration detection) OR (vibration detection threshold) OR (VDT) OR (vibration testing) OR (vibration sensitivity))
